# Prevalence and risk factors of gastroesophageal reflux symptoms in a Chinese retiree cohort

**DOI:** 10.1186/1471-230X-12-161

**Published:** 2012-11-15

**Authors:** Tiantian Chen, Ming Lu, Xiaofeng Wang, Yajun Yang, Juan Zhang, Li Jin, Weimin Ye

**Affiliations:** 1 Clinical Epidemiology Unit, Qilu Hospital of Shandong University, Jinan, 250012, China; 2 Department of Epidemiology and Health Statistics, School of Public Health, Shandong University, Jinan, 250012, China; 3 MOE Key Laboratory of Contemporary Anthropology, School of Life Sciences, Fudan University, Shanghai, 200433, China; 4 China Medical City Institute of Health Sciences, Taizhou, Jiangsu, 225300, China; 5 Department of Medical Epidemiology and Biostatistics, Karolinska Institutet, Stockholm, 17177, Sweden

**Keywords:** Gastroesophageal reflux symptoms, Body mass index, Obesity, Abdominal obesity, Retired population

## Abstract

**Background:**

Data about prevalence of gastroesophageal reflux diseases (GERD) from Asian populations are still scarce. To provide additional data on prevalence of GERD and investigate its potential risk factors, we performed this cross-sectional study in the Taizhou Retiree Cohort.

**Methods:**

After physical examination, the participants were asked whether they suffered with heartburn or acid regurgitation in the last 12 months by trained interviewers, and if yes, the severity and frequency of the symptoms were recorded. Odds ratios (ORs) with 95% confidence intervals (CIs) for the associations of obesity and other risk factors with GERD were derived from logistic regression models.

**Results:**

8831 retirees completed the questionnaire and physical examination. In total 150 (1.7%) reported the symptoms occurring at least once per week within the last 12 months before the interview. Compared with subjects without GERD, having a history of diabetes mellitus (OR 2.2, 95% CI 1.4-3.5), hypertension (OR 1.4, 95% CI 1.0-2.1), gastritis (OR 8.2, 95% CI 5.8-11.5), peptic ulcer (OR 3.3, 95% CI 1.8-6.1) and high triglyceride level (≥1.81mmol/L) (OR 2.0, 95% CI 1.2-3.4) were associated with a significantly increased risk of GERD. However, there was no significant association between body mass index, waist-to-hip ratio or waist alone, smoking, consumption of alcohol & tea, and the occurrence of reflux symptoms.

**Conclusions:**

Compared with Western populations, the prevalence of GERD in this Chinese retiree cohort is low. A history of diabetes mellitus, hypertension, gastritis, peptic ulcer or hypertriglyceridaemia increases GERD risk in this population.

## Background

Gastroesophageal reflux disease (GERD) is a chronic disease that is associated with a range of troublesome symptoms, which can in turn have a significant impact on health-related quality of life. Western countries have experienced GERD epidemic in recent decades; a systematic review article concluded that the prevalence of GERD was 10-20% in Western countries [1]. Recently, the prevalence of GERD has been reported to increase rapidly in Asia [2-5]. However, studies on Asian populations are still scarce.

There is a widespread notion that obesity and abdominal obesity may increase the risk of GERD, especially in Western populations [6-9]. High body mass index (BMI) is associated with a dose-dependently increased risk for GERD, erosive esophagitis, and esophageal adenocarcinoma. Several studies have reported that the adverse effect of obesity on GERD is through mechanical alterations at the esophagogastric junction [10,11]. However, since not every obese patient develops GERD, the pathogenesis must be multifactorial and cannot be explained by a single physiological parameter. Additional observations demonstrated the association between GERD and metabolic risk factors including hypertension, hypertriglyceridaemia and diabetes mellitus [12-14]. There is also suggestive relationship between GERD and peptic ulcer and gastritis [15,16], but the results are not consistent.

In this study, we therefore intended to investigate the prevalence of gastroesophageal reflux symptoms and to determine whether BMI, waist circumference, waist-to-hip ratio and other factors are associated with the symptoms in the Taizhou Retiree Cohort.

## Methods

### The study cohort and data collection

Taizhou Longitudinal Study phase II (retiree cohort) was started from December of 2007, detailed description of study methods has been published previously [17], Briefly, all retirees who registered in the Social Insurance Bureau of Hailing (a district of Taizhou city), were invited to undergo a medical examination and their blood samples were collected for testing serum lipid, glucose and other biochemical profile. Information on demographic characteristics, lifestyle, as well as history of major chronic diseases was also collected for each participant. A simplified questionnaire about gastroesophageal reflux symptoms was added since September of 2008 and used till the end of enrollment, August 2009 [18,19]. The trained interviewers asked the participants whether or not they suffered with heartburn or acid regurgitation in the past 12 months, and if yes, the frequency of the symptoms, and the age the symptoms started. Heartburn and acid regurgitation are the cardinal symptoms of gastroesophageal reflux disease, and the use of questionnaires to assess these symptoms is well validated as a reliable measurement of the true occurrence of reflux [20,21]. Symptom of “heartburn” was defined as “the burning sensation behind breast bone”, and “acid regurgitation” was defined as “a bitter or sour tasting fluid coming into the throat or chest”. Symptom frequency was reported as: less than once per month, once per month, once per week, 2-3 times per week, 3-4 times per week or daily. We also asked whether the symptoms affected subjects’ daily life including sleep, dietary habits and physical activity. The influences of the of symptoms were reported as negligible, mild (almost can be ignored), moderate (cannot be ignored but can be tolerated), severe (affects normal daily life) and very severe (markedly, affects normal daily life). A summary severity score was then created by taking max value of the influences, and grouping negligible and mild category together. Participants were also asked whether they took medication for their symptoms, such as H2-receptor antagonist (H2Ra) or proton pump inhibitors (PPI).

### Exposure assessment

Body weight, height, waist and hip circumference were measured by trained staff. The waist circumference was measured midway between the caudal point of the costal arch as palpated laterally and the iliac crest. BMI (kg/m^2^) was calculated as weight (kg) divided by square of height (m). WHR was defined as waist divided by hip circumference. Further, we categorized BMI into three groups, <24.0, 24.0-27.9 and ≥28.0. Overweight was defined as BMI between 24.0 and 27.9, and obesity was defined as BMI ≥28.0 according to the definition proposed by Working Group on Obesity in China [22].

Abdominal obesity was defined as women with a waist of ≥ 88 cm or man with a waist of ≥ 92 cm (both were upper quarter of waist accordingly). Women with a WHR of ≥ 0.89 or man with a WHR of ≥ 0.95 were also defined as central obesity.

Smoking status was categorized into three groups: ever smoked but quit now, current smokers and never smokers. Participants who reported an average frequency of alcohol intake of over 4 times per month were regarded as regular drinkers; tea drinkers were defined as at least one cup per day.

Participants were also asked to report their history of diseases (with diagnosis from a hospital), including diabetes mellitus, hypertension, cardiovascular and cerebrovascular diseases, tumor, asthma, gastritis, peptic ulcer, hepatitis, tuberculosis and gallstone. Medications taken by participants regularly were also recorded.

### Statistical analysis

We defined GERD cases as ever experiencing symptoms of heartburn and/or regurgitation at least once per week. The control group comprised of those without reported GERD.

Odds ratios (ORs) with 95% confidence intervals (CIs) for the association of potential risk factors with GERD were derived from logistic regression models. All analyses were conducted using SAS version 9.1 (SAS Institute Inc, Cary, NC). Two-sided *P* values <0.05 were considered statistically significant. The study was approved by the Human Ethics Committee of Fudan University. Written informed consent was obtained from all participants.

## Results

From August 2008 to the end of baseline enrollment (Aug 2009), 8867 retirees aged 40 to 93 years completed the questionnaire and physical examination. In the procedure of data cleaning, we deleted 36 records for incomplete age, weight or height. Cohort demographic characteristics are presented in Table 1. Among 8831 participants (with a mean age of 62.5±8.3 years), 64.9% were females and females tended to have lower education level (primary school or lower, female: 60.7% vs. male: 41.4%), and more likely to be blue collar workers (93.9% vs. 79.6%).

Among these 8831 participants, only 158 (1.8%) reported ever having symptom of heartburn, 907 (10.3%) reported ever having symptom of acid regurgitation. Among all these symptomatic subjects, 499 (5.7%) had reflux symptom less than once per month, 281 (3.2%) reported once per month, 72 (0.8%) once per week, 55 (0.6%) several times per week, and 23 (0.3%) daily (Table 1). For the age group 40-49, 50-59, 60-69, 70-79, and 80+, the prevalence rates were 12.8%, 11.6%, 10.2%, 8.8%, and 10.0%, respectively. For women, the prevalence in each age group was: 12.4%, 11.2%, 11.9%, 10.8% and 9.9%. For men, the prevalence was: 14.8%, 14.8%, 8.3%, 7.2% and 10.2%, respectively.

**Table 1:** Characteristics of the Taizhou Retiree Cohort

	**Male (n=3097)**	**Female (n=5734)**
Age (years, mean±SD)	66.48±6.91	60.36±7.61
BMI (mean±SD)	24.53±3.22	24.89±3.51
Waist (cm, mean±SD)	86.30±8.71	82.72±8.71
WHR (mean±SD)	0.91±0.06	0.85±0.05
SBP (mmHg, mean±SD)	141.4±19.7	138.9±20.3
DBP (mmHg, mean±SD)	88.3±12.1	83.3±11.5
Education (%)		
Primary school or lower	1283 (41.4)	3478 (60.7)
Primary high school	1045 (33.8)	1535 (26.8)
Secondary high school	551 (17.8)	663 (11.6)
College and above	217 (7.0)	55 (0.9)
Occupation (%)		
Blue collar	2463 (79.6)	5380 (93.9)
White collar	632 (20.4)	345 (6.1)
Smoking (%)		
Ex-smokers	598 (19.3)	36 (0.6)
Current smokers	1475 (47.7)	293 (5.1)
Never	1019 (33.0)	5403 (94.3)
Alcohol drinking (%)		
Yes	1208 (39.0)	97 (1.7)
No	1889 (61.0)	5637 (98.3)
Tea drinking (%)		
Yes	1570 (50.7)	537 (9.4)
No	1526 (49.3)	5159 (90.6)
Frequency of gastroesophageal reflux symptoms		
Never	2820 (91.1)	5081 (88.6)
Less than once per month	156 (5.0)	343 (6.0)
Once per month	71 (2.3)	210 (3.7)
Once per week	22 (0.7)	50 (0.9)
Several times per week	15 (0.5)	40 (0.7)
Daily	13 (0.4)	10 (0.2)

BMI: body mass index (weight[kg]/height^2^[m]); WHR: waist-to-hip ratio; SBP: systolic blood pressure; DBP: diastolic blood pressure.

For further analyses, we defined the 150 (1.7%) participants who reported the symptoms (either heartburn or acid regurgitation) at least once a week as GERD cases. Severity and frequency of the symptoms among the 150 cases and the medications they took are presented in Table 2. We deleted 780 participants who reported having the symptom once per month or less to avoid bias from misclassification. The rest of 7901 (89.5%) participants, who reported having neither heartburn nor acid regurgitation, as well as not taking any anti-acids, were considered as controls.

**Table 2:** Severity, use of medication, and frequency of symptoms among subjects with at least once per week gastroesophageal reflux episode

	**Frequency of the gastroesophageal reflux symptoms**			**Total (%)**
	**Once per week (%)**	**Several times per week (%)**	**Daily (%)**	
Severity^a^				
Negligible or Mild	65 (90.2)	49 (89.1)	16 (69.6)	130 (86.7)
Moderate	2 (2.8)	1 (1.8)	2 (8.7)	5 (3.3)
Severe	2 (2.8)	1 (1.8)	1 (4.3)	4 (2.7)
Very severe	3 (4.2)	4 (7.3)	4 (17.4)	11 (7.3)
Medication use				
None	50 (69.4)	36 (65.4)	10 (43.5)	96 (64.0)
H2Ra	17 (23.6)	14 (25.5)	9 (39.1)	40 (26.7)
PPI	4 (5.6)	4 (7.3)	4 (17.4)	12 (8.0)
Other anti-acid medication	1 (1.4)	1 (1.8)	0 (0.0)	2 (1.3)

H2Ra: H2-receptor antagonist; PPI: proton pump inhibitors.

^a^Mild: almost can be ignored; Moderate: cannot be ignored but can be tolerated; Severe: affects normal daily life; Very severe: markedly, affects normal daily life.

### Obesity related factors

Compared with subjects with normal weight, overweight subjects (BMI 24.0-27.9 kg/m^2)^ had a multivariable-adjusted OR of 1.0 (95%CI 0.7-1.4) for developing the symptoms and the corresponding OR was 0.7 (95%CI 0.4-1.2) for obese subjects (BMI ≥28.0 kg/m^2^) (Table 3). Similarly, waist-to-hip ratio or waist alone did not show any significant association with the symptoms (Table 3).

**Table 3:** Obesity related factors and risk of symptomatic gastroesophageal reflux

**Variables**	**Cases**	**Controls**	**Unadjusted OR**	**95% CI**	**Adjusted OR**^**a**^	**95% CI**
BMI (kg/m^2^)						
<24.0	66	3363	1.0	(ref)	1.0	(ref)
24.0-27.9	65	3251	1.0	(0.7-1.4)	1.0	(0.7-1.4)
≥28.0	19	1287	0.8	(0.5-1.3)	0.7	(04.-1.2)
Waist^b^ (cm)						
Low	116	6021	1.0	(ref)	1.0	(ref)
High	34	1879	0.9	(0.6-1.4)	0.9	(0.6-1.3)
WHR^c^						
Low	106	5915	1.0	(ref)	1.0	(ref)
High	44	1985	1.2	(0.9-1.8)	1.1	(0.8-1.6)

BMI: body mass index (weight[kg]/height^2^[m]); WHR: wasit-to-hip ratio; OR, odds ratio; CI, confidence interval.

^a^In the multivariate logistic regression model adjustments were made for age, sex, education, occupation, tobacco smoking, alcohol drinking, tea drinking, history of diabetes mellitus, hypertension, gastritis or peptic ulcer, and serum TG.

^b^Low: Male<92cm and female<88cm; High: Male≥92cm and female≥88cm.

^c^Low: Male<0.95 and female<0.89; High: Male≥0.95 and female≥0.89.

### Lifestyle related factors

Smoking was not associated with any change in the risk of GERD. There was also no significant association between alcohol drinking, tea drinking and the occurrence of reflux symptoms (Table 4).

**Table 4:** Lifestyle related factors and risk of symptomatic gastroesophageal reflux

**Variables**	**Cases**	**Controls**	**Unadjusted OR**	**95% CI**	**Adjusted OR**^**a**^	**95% CI**
Smoking						
Never	108	5739	1.0	(ref)	1.0	(ref)
Ex-smokers	16	559	1.5	(0.9-2.6)	1.5	(0.8-3.1)
Current smokers	26	1597	0.9	(0.6-1.3)	0.9	(0.5-1.6)
Alcohol drinking						
Never	129	6713	1.0	(ref)	1.0	(ref)
Regular	21	1188	0.9	(0.6-1.5)	1.2	(0.7-2.1)
Tea drinking						
No	120	5985	1.0	(ref)	1.0	(ref)
Yes	30	1914	0.8	(0.5-1.2)	0.8	(0.5-1.3)

OR, odds ratio; CI, confidence interval.

^a^ORs were mutually adjusted for variables listed in the table, as well as age, sex, waist-to-hip ratio, history of diabetes mellitus, hypertension, gastritis, or peptic ulcer, and serum TG.

### Past history of diseases

Subjects with self-reported diabetes mellitus were more likely to have report the symptoms, with a multivariable-adjusted OR of 2.2 (95% CI 1.4-3.5). Hypertension was also strongly associated with the development of the symptoms and the corresponding OR was 1.4 (95% CI 1.0-2.1) (Table 5).

**Table 5:** Past history of diseases and risk of symptomatic gastroesophageal reflux

**Variables**	**Cases**	**Controls**	**Unadjusted OR**	**95% CI**	**Adjusted OR**^**a**^	**95% CI**
Diabetes Mellitus						
No	122	7134	1.0	(ref)	1.0	(ref)
Yes	28	766	2.1	(1.4-3.2)	2.2	(1.4-3.5)
Hypertension						
No	84	5067	1.0	(ref)	1.0	(ref)
Yes	66	2831	1.4	(1.0-1.9)	1.4	(1.0-2.1)
Gastritis						
No	85	7240	1.0	(ref)	1.0	(ref)
Yes	65	655	8.5	(6.1-11.8)	8.2	(5.8-11.5)
Peptic ulcer						
No	134	7719	1.0	(ref)	1.0	(ref)
Yes	16	181	5.1	(3.0-8.7)	3.3	(1.8-6.1)
Serum TG						
<1.81mmol/L	131	7378	1.0	(ref)	1.0	(ref)
≥1.81mmol/L	19	523	2.0	(1.3-3.3)	2.0	(1.2-3.4)

OR, odds ratio; CI, confidence interval.

^a^ORs were mutually adjusted for variables listed in the table, as well as age, sex, waist-to-hip ratio, tobacco smoking, alcohol drinking, and tea drinking.

Seven hundred and twenty participants reported having chronic gastritis. Compared with those without, subjects with chronic gastritis have an unadjusted OR of 8.5 (95% CI 6.1-11.8) to report GERD symptom. This relation remained stable after further adjustment for all covariates, with an OR of 8.2 (95% CI 5.8-11.5) (Table 5).

Similarly, retirees who had a history of peptic ulcer tended to be more prone to report the reflux symptoms, with an unadjusted OR of 5.1 (95% CI 3.0-8.7), and the association remained strong after multivariable-adjustment, with an OR of 3.3 (95% CI 1.8-6.1) (Table 5).

High serum TG (≥1.81mmol/l) was significantly associated with the presence of GERD (OR: 2.0; 95% CI: 1.3-3.3). This relation remained stable after further adjustment for all covariates, with an OR of 2.0 (95% CI 1.2-3.4) (Table 5).

## Discussion

This population-based study on the epidemiology of GERD surveyed a total of 8867 retired individuals in China. The overall prevalence of GERD in the present study was 1.7% which was lower than that in previous Chinese population-based surveys using a variety of definitions of GERD. A possible explanation for this difference is that the participants in this study were all retirees who did not have much psychological stress. The occurrence and response to treatment of GERD were reported to be associated with stress [23]. In addition, the study subjects in several previous studies were patients who underwent endoscopy in medical centers or those who attended health screening. It is well known that subjects with gastrointestinal symptoms are more likely to go for this kind of examination.

A clear association between GERD and obesity has been found in Western countries [6,9]. But we did not find an association between obesity and GERD. Previous population-based studies on the association between GERD and BMI in China have also reported inconsistent results [16,24]. In this study, we assessed the association between GERD and abdominal obesity (indicated by waist circumference and waist-to-hip ratio), but the results did not show any significant association between central obesity and the reflux symptoms. This could potentially be explained by relatively low prevalence of obesity in China.

In the current study, we found an association between diabetes mellitus and GERD. This is consistent with the previous studies performed in Japan [13] and Korea [25,26]. A Korean study reported that about 23.1% of the patients with type II diabetes experienced at least weekly typical GERD symptoms [25]. Another study showed that the prevalence of GERD symptoms in diabetes patients was 41% [14], which was higher than the reported prevalence in the general population. There are several possible mechanisms underlying diabetes-related GERD. Some studies have shown that many gastrointestinal symptoms in diabetes patients are linked to motor dysfunction and that the neuropathy in diabetes patients may play an important role on the development of GERD symptoms. Autonomic nervous disorder in diabetes patients might cause a certain degree of gastric dysmotility which may increase intragastric distension and lead to a higher incidence of transient lower esophageal sphincter relaxation [25].

Among the individual components of metabolic syndrome, an elevated level of serum TG was a significant predictive factor for the GERD. This result is consistent with previous studies about metabolic syndrome and GERD or erosive esophagitis [12,27,28]; however, a Japanese study did not find such an association between elevated serum TG and erosive esophagitis [13]. It has been reported that hypertriglyceridemia is associated with increased insulin resistance [29] and chronic *Helicobacter pylori* infections which was suggested to be a protective factor for erosive esophagitis can modify the serum lipid profile [30,31]. Therefore, elevated serum TG levels may be just an epiphenomenon accompanying other etiologic factors.

Hypertension was found, after adjusting for BMI, to be associated with GERD. In a Japanese study, hypertension and hyperglycaemia were independent risk factors for erosive esophagitis [13]. In a Korean population, the presence of metabolic syndrome and a higher visceral adipose tissue area were suggested as risk factors for esophagitis [27]. Consistent with the above, we found hypertension was a risk factor for symptom-defined GERD, but we did not investigate drug therapy of the participants in the present study. Calcium antagonists used to treat hypertension decrease the lower esophageal sphincter pressure and inhibit muscle contraction in the esophagus itself [32]. In China, calcium antagonists are widely used to treat hypertension, so it is possible that antihypertensive therapy might explain the findings in our study.

The current study supports an association of GERD with a history of peptic ulcer disease and gastritis. A large population-based study on the epidemiology of GERD across five regions in China also reported an association between GERD and peptic ulcer and gastritis [16]. In contrast, a study performed in Finland found a protective effect for gastritis against GERD [15]. However, it is important to distinguish between clinically diagnosed gastritis and the patient-reported history of gastritis which was used in the current study. In China, “gastritis” generally indicates upper gastrointestinal discomfort and a diagnosis is unlikely to be based on endoscopic examination biopsy or serum pepsinogen measurement. The major limitation of our study is that the medical history of participants was self-reported is the biggest limitation of our study, but it should be noted that it is a standard practice in China for patients to keep their own medical records which, in some extent, may reduce the impact of this limitation.

## Conclusion

This study showed the prevalence of GERD in this retiree cohort is lower than that in Western populations, and its occurrence is independent of obesity or abdominal obesity. However, elevated level of serum TG, a medical history of hypertension, diabetes, peptic ulcer disease and gastritis are associated with an increased risk of developing GERD in this population. Further studies are needed to more fully elucidate potential mechanisms underlying these relationships.

## Competing interests

The authors declare that they have no competing interests.

## Authors’ contributions

ML, LJ, WY, XW and YY made substantial contributions to the conception and design of the study. TC and ML performed the statistical analysis and drafted the manuscript. WY, ML and TC have been involved in revising the manuscript critically for important intellectual content. JZ participated in data collection. All authors read and approved the final manuscript.

## Pre-publication history

The pre-publication history for this paper can be accessed here:

http://www.biomedcentral.com/1471-230X/12/161/prepub
